# Normal Hearing Ability but Impaired Auditory Selective Attention Associated with Prediction of Response to Donepezil in Patients with Alzheimer's Disease

**DOI:** 10.1155/2015/540348

**Published:** 2015-06-16

**Authors:** Yoshitaka Ouchi, Kenichi Meguro, Kyoko Akanuma, Yuriko Kato, Satoshi Yamaguchi

**Affiliations:** Division of Geriatric Behavioral Neurology, CYRIC, Tohoku University, 4-1 Seiryo-machi, Aoba-ku, IDAC, Sendai 980-8575, Japan

## Abstract

*Background.* Alzheimer's disease (AD) patients have a poor response to the voices of caregivers. After administration of donepezil, caregivers often find that patients respond more frequently, whereas they had previously pretended to be “deaf.” We investigated whether auditory selective attention is associated with response to donepezil.* Methods. *The subjects were40 AD patients, 20 elderly healthy controls (HCs), and 15 young HCs. Pure tone audiometry was conducted and an original Auditory Selective Attention (ASA) test was performed with a MoCA vigilance test. Reassessment of the AD group was performed after donepezil treatment for 3 months.* Results. *Hearing level of the AD group was the same as that of the elderly HC group. However, ASA test scores decreased in the AD group and were correlated with the vigilance test scores. Donepezil responders (MMSE 3+) also showed improvement on the ASA test. At baseline, the responders had higher vigilance and lower ASA test scores.* Conclusion.* Contrary to the common view, AD patients had a similar level of hearing ability to healthy elderly. Auditory attention was impaired in AD patients, which suggests that unnecessary sounds should be avoided in nursing homes. Auditory selective attention is associated with response to donepezil in AD.

## 1. Introduction

Patients with Alzheimer's disease (AD) often respond poorly to the voices of family members or caregivers at home and in outpatient clinics and nursing homes. Speaking loudly to the patients is not always successful and this places a burden on caregivers. Alternatively, hearing loss may be considered to be part of the “normal aging” process and may not be thought to be important by caregivers. Reports on the positive effects of psychosocial intervention [1] indicate that music is common at nursing homes and clinics [2]; however, the appropriate loudness for patients is unclear.

After administration of cholinesterase inhibitors (ChEIs) such as donepezil, family members, and caregivers often find that AD patients became more responsive to their voices. They may feel that the patient had previously pretended to be “deaf.” Clinical studies of the effect of donepezil [3–5] show that psychomotor attention or mental speed is activated through increased cerebral blood flow in the frontal lobes. However, to date, there is no evidence that hearing impairment (an ear problem) is present in addition to auditory attention disability (a brain problem) in AD patients or whether hearing impairment itself improves after ChEI administration.

Not all AD patients have a good response to ChEIs; 26–63% reported to be responders [6]. We have shown that good responders can be predicted by positron emission tomography (DNP-PET) in a study using [^11^C] donepezil [7]. However, DNP-PET requires use of specific instruments at research centers and a more clinically practical method for prediction of responders is needed.

In the current study, we hypothesized that (1) hearing ability in AD patients is impaired compared with healthy controls, (2) auditory attention is also deteriorated, and (3) drug responders can be detected by cognitive tests assessing auditory selective attention. Several reports have examined visual selective attention [8, 9], but auditory selective attention has not been fully investigated in AD patients. Thus, we developed an original auditory selective attention (ASA) task as part of the study.

## 2. Methods

### 2.1. Patients

The subjects were consecutive outpatients with characteristics consistent with the following entry and exclusion criteria. All were outpatients at the memory clinic of the SKIP Center, an integrated institute for stroke, dementia, and bed-confinement prevention in Tajiri, Miyagi prefecture, northern Japan [10]. Two neurologists, one psychiatrist, one laryngologist, and one ophthalmologist work at the center.

The entry criteria were (1) probable AD based on the NINCDS-ADRDA criteria [11] and (2) a minimental state examination (MMSE) [12] score ≥9 to ensure understanding of instructions. The exclusion criteria were (1) the presence of cerebrovascular diseases shown by MRI (1.5 T), (2) the presence of laryngological diseases as diagnosed by a laryngologist, and (3) previous administration of donepezil or another ChEI. An elderly healthy control (HC) group was recruited from age-matched caregivers of the AD patients and a young HC group was formed from staff members at the SKIP Center as well as from volunteers. They showed no cognitive impairment based on the clinical observations. The exclusion criteria were (1) the presence of laryngological diseases as diagnosed by a laryngologist and (2) previous administration of donepezil or another ChEI.

Demographics of the study population were shown in Table 1.

### 2.2. Sample Size Determination

In the present study, the pre-and postadministration were calculated to be 14.3 cases, which was based on the power calculation. The rate of occurrence of improvement for donepezil group was 0.5. The error protection was 0.05 and power was 0.8. The sample size of donepezil administration adjusted to 15 cases. Finally, 40 AD patients, 20 elderly HCs, and 15 young HCs participated in the study; 15 AD patients from among the 40 patients also agreed to participate in the reassessment after the donepezil treatment (see below). This was a clinical study using the consecutive outpatients; all patients completed the study. For the 25 patients (40 minus 15) not participated in the follow-up study, 6 did not tolerate donepezil administration due to side effects, 8 did not want to be tested with audiometry again, and 11 wanted to take other drugs due to economical reasons.

The period of this study was from July 2013 to June 2014. Written informed consent was obtained from all HC adults and from the patients themselves, together with their families. This study was approved by the Ethical Committee of Tohoku University Graduate School of Medicine.

### 2.3. Assessments

All assessments below were performed blindly to the condition of the study.

#### 2.3.1. Pure Tone Audiometry

Pure tone audiometry, including air conduction thresholds at 0.125–8 kHz and bone conduction thresholds at 0.5–4 kHz, was performed using a GN Resound Orbiter 922 version 2 audiometer, according to ISO 8253-1 [13], using Telephonics TDH-39 earphones and a Radio Ear B71 bone conductor in a sound-attenuating booth complying with standards specified in ISO 8253-2 [14].

#### 2.3.2. Auditory Selective Attention (ASA) Test

To prepare a valid assessment tool, we made a preliminary assessment of the loudness in a hall of a typical nursing home with a television. The loudness was found to be 60–65 dB. When group activities such as exercise were performed, the loudness increased to 70–80 dB. Thus, the noise level was set at 60 dB and 70 dB (see below).

Patients were seated in front of a computer screen and were asked to push a button immediately after hearing a target voice among the noise. The noise was made by summing 10 women's voices reading aloud newspapers at normal speed. The target sentence “please push the button” was spoken by another woman and was presented during the noise. The target sentence was presented 15 times during 270 s of noise.

In a preliminary experiment in which the noise and target voice were presented at the same loudness of 60 dB, we found that all patients were able to respond correctly. Then, in Condition 1, the target voice and noise were presented at 55 dB and 60 dB, respectively. In Condition 2, these levels were 70 dB and 65 dB, respectively.

#### 2.3.3. Vigilance Test (Modified MoCA-J)

A modified version of the vigilance test of the Montreal Cognitive Assessment (MoCA) [15] was used as an auditory selective attention test. The original vigilance test uses one kana “A” as the target, whereas we used “Shi,” “U,” and “Ke.” These three sounds were each presented 11 times; thus, the scores ranged from 0 to 33.

#### 2.3.4. Questionnaire on Auditory Attention in Daily LIFE (Auditory ADL)

We prepared an original questionnaire as follows.

- Response to one's own name being called:
  1. How does the patient respond to his/her name being called at silence?
  2. How does the patient respond to his/her name being called when a TV or radio is turned on?
  3. How does the patient respond to his/her name being called under other voice?
  4. How does the patient respond to his/her name being called in a waiting room at a hospital?
- Listening to other people talk under conditions:
  1. How does the patient respond to other people talk at silence?
  2. How does the patient respond to other people talk when a TV or radio is turned on?
  3. How does the patient respond to other people talk under other voice?
  4. How does the patient respond to other people talk in a waiting room at a hospital?

The answers were scored as follows: 1, an immediate reaction; 2, two repetitions of the question are needed; 3, three or more repetitions are needed; and 4, another stimulation is needed, such as tapping of the shoulder. The best score was 8 (a score of 1 for each question) and the worst score was 32 (a score of 4 for each question). There was perfect test-retest reliability for the same caregivers.

## 3. Analyses

All analyses below were performed blindly to the condition of the study. All analyses below used the patients' age and educational level as covariance. Time between the onset of dementia and treatment initiation was not considered, since the information of dementia onset was variable due to families.

### 3.1. Cross-Sectional Group Analysis

Differences among the young HC, elderly HC, and AD groups were analyzed using one-way analysis of variance (ANOVA).

### 3.2. Clinical Validity of the ASA Test

The ASA test was repeated for 20 patients at 2-week intervals for assessing test-retest reliability. The ASA test was clinically validated using the MoCA vigilance test and the questionnaire on auditory attention in daily life.

### 3.3. Longitudinal Analysis after Donepezil Administration in the AD Group

After assessments at baseline, 5 mg/day of donepezil was administered to the AD patients. In Japan, donepezil administration begins with 3 mg/day and keeps with 5 mg/day. After three months, 15 patients agreed to participate in the reassessment to evaluate the effect of donepezil.

### 3.4. Predicting Donepezil Responders

Donepezil responders were defined as patients with an increase in MMSE score ≥3 after treatment. We defined an “improved case” as that showed three or more increment in MMSE score, and all of the remaining cases were defined as an “unimproved case.” The improvement of MMSE was three points or more increase because it has been defined as limitation of aberrant changes in many clinical trials [16].

Combinations among the MMSE, ASA, and vigilance tests were examined to find a method for identification of responders at baseline.

## 4. Results

### 4.1. Cross-Sectional Group Analysis

**Figure 1:**
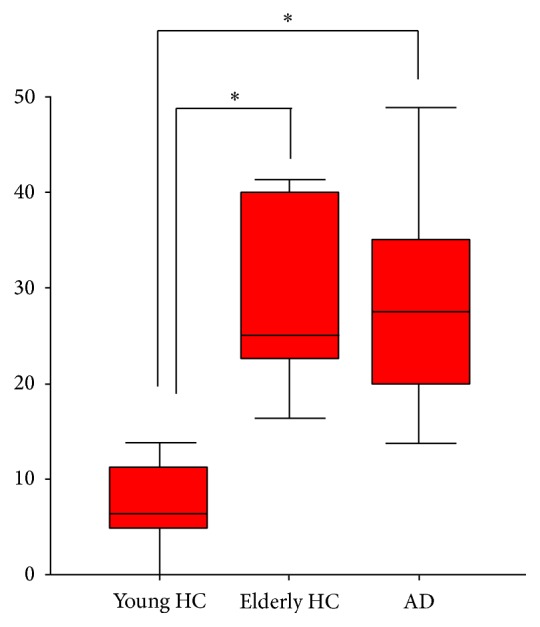
Hearing level of three groups. HC: healthy control; AD: Alzheimer's disease. There is a significant group effect (one-way ANOVA, *P* < 0.01). A post hoc test indicated the group differences (^*^ *P* < 0.05).

As shown in Figure 1, the hearing level in the AD group was about the same as that in the elderly HC group, and both were significantly impaired compared to that of the young HC group. The mean hearing thresholds in all 3 groups did not exceed 20 dB HL at any frequency between 0.125 and 2 kHz in any ear. A mean threshold elevation at 3–8 kHz, not greater than 50 dB HL in any ear, was found in all three groups. There were no significant between-group differences in hearing threshold levels at any frequency or in any ear, and no significant interaural differences; thus mean results for the right-left ears were used.

**Figure 2:**
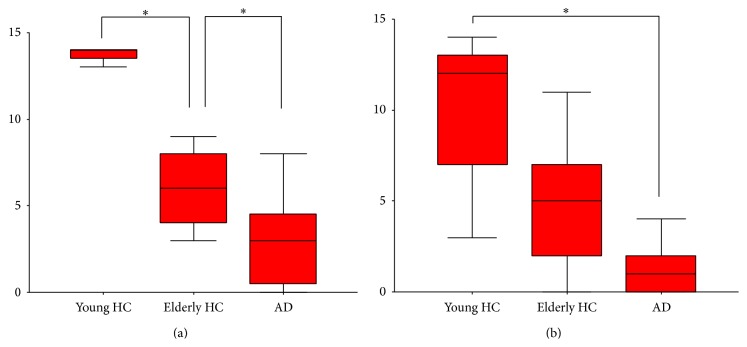
ASA task results under the 60 db (a) and 70 db (b) conditions for three groups. HC: healthy control; AD: Alzheimer's disease. There is a significant group effect (one-way ANOVA, *P* < 0.01) for both conditions. A post hoc test indicated the group differences (^*^ *P* < 0.05).

Ability on the ASA test decreased from the young HC to elderly HC group and then to the AD group, but the difference between the elderly HC and AD groups was not significant for the 70 dB condition (Figure 2). ASA test scores had no significant correlation with MMSE scores for the 60 dB (Spearman *r* = −0.12) and 70 dB (*r* = −0.31) conditions.

### 4.2. Clinical Validity of the ASA Test

Repetition of the ASA test for 20 patients at 2-week intervals showed good test-retest reliability. ASA test scores were significantly correlated with vigilance test scores for the 60 dB condition (*r* = −0.61, *P* < 0.01), but not for the 70 dB condition (*r* = 0.43), and were also significantly correlated with the questionnaire scores.

### 4.3. Longitudinal Analysis after Donepezil Administration for the AD Group

Changes in variables after donepezil treatmentare shown in Table 2. Donepezil responders (*n* = 6 versus 9 nonresponders) were defined as patients for whom MMSE scores increased by ≥3 after treatment. Responders had improved ASA (60 dB condition) and questionnaire scores, but no significant changes in hearing level and vigilance test scores.

### 4.4. Predicting Donepezil Responders

**Figure 3:**
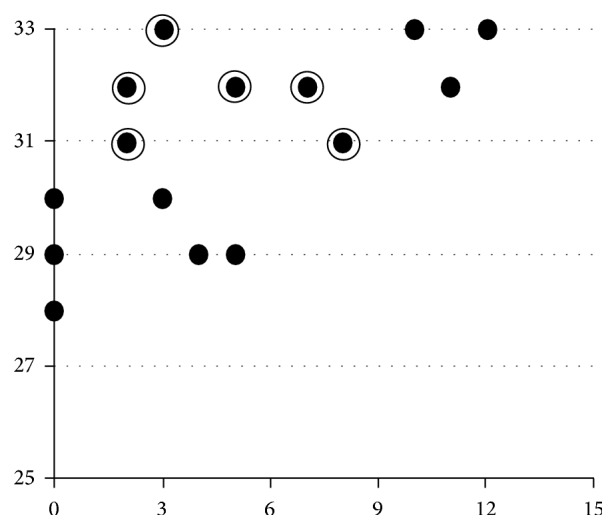
Vigilance (MoCA) and ASA task. ASA: auditory selective attention. The horizontal line shows the ASA task (60 db) scores and the vertical line indicates the vigilance test scores. For both tests, higher scores mean better performances. There was a significant Spearman's correlation (Rs = 0.611, *P* < 0.01) between the scores. Double circles mean donepezil responders.

Figure 3 illustrates the relationship of ASA test and vigilance test scores (plotted as double circles) with response to donepezil. At baseline, donepezil responders had higher scores on the vigilance test and lower scores on the ASA test.

## 5. Discussion

In performing the study, we hypothesized that (1) hearing ability in AD patients is impaired compared with healthy controls, (2) auditory attention is deteriorated, and (3) responders to donepezil can be detected by cognitive tests of auditory attention. Our findings support the second and third hypotheses, but contrary to general belief, AD patients showed normal hearing ability, and thus the first hypothesis was not proved. Before discussing the results, we address some methodological issues.

### 5.1. Methodological Issues

We found good test-retest reliability for the ASA test. This test was also clinically validated by the MoCA vigilance test and the questionnaire. Other attention tests such as the dichotic digits test [17] may have been more appropriate, but the time limitation for dementia patients prevented use of sophisticated neuropsychological tests. A similar reason has been given for the incomplete performance of functional neuroimaging. The external validity was not considered; that is, the results of this study cannot be extrapolated to patients with a MMSE score <9. Also, the small populations and short follow-up period with losses of follow-up were limitations. Despite these limitations, we consider that the results allow a better understanding of the neurophysiology in AD and treatment for patients.

### 5.2. Hearing Ability and Auditory Attention

In contrast to a common perception, AD patients had the same level of hearing ability as that of healthy elderly controls. Thus, caregivers should be instructed that AD patients do not have impaired hearing ability. Instead, auditory attention may be impaired in a noisy environment, and unnecessarily sounds should be avoided in a nursing home for AD patients.

The Baltimore Longitudinal Study of Aging examined whether hearing loss is associated with incident AD [18]. Compared with normal hearing, the risk of incident AD increased with baseline hearing loss (HR 1.20 per 10 dB of hearing loss), which suggests that hearing loss is independently associated with dementia. Whether hearing loss is a marker for early-stage dementia or is actually a modifiable risk factor for dementia deserves further study.

To clarify the mechanism of the central auditory dysfunction in AD, Gates et al. [19]examined 313 volunteers from the Adult Changes in Thought surveillance cohort with adequate peripheral hearing. The composite executive function score was found to be significantly associated with each central auditory measure, and the Trail Making Test B was most strongly associated with auditory outcomes. In elderly persons, reduced executive function is associated with central auditory processing, but not with primary auditory functions. This suggests that central presbycusis and executive dysfunction may result from similar neurodegenerative processes.

### 5.3. Identification of Donepezil Responders

Three ChEIs (donepezil, galantamine, and rivastigmine) are used in AD. All are symptomatic, rather than curative, and the choice of each drug mainly depends on clinicians' experiences. Evidence-based drug choice is likely to improve outcomes, but clinically practical methods of predicting drug responders have not been established. The frontal association cortex was found to be activated in a cocktail party condition using functional MRI [20] and donepezil treatment increases blood flow in the frontal lobe [3–5]. Our results (Figure 3) show that donepezil responders had higher scores on a vigilance test and lower scores on the ASA task before treatment. This suggests that those with higher “simple auditory attention” but lower “complex” auditory selective attention have a residual capacity to be stimulated by the drug, so as to increase “complex” attention. Some of these responders were previously assessed by donepezil PET [7] and were found to have higher acetylcholinesterase activities (data not shown).

The combination of a MoCA-J vigilance test and the auditory selective attention task at baseline may allow detection of probable donepezil responders, who could then be administered donepezil, with nonresponders treated with other ChEIs. The acetylcholine concentration after donepezil treatment has been found to be higher than that after treatment with other ChEIs [21]. Thus, attention ability may be improved more by donepezil compared to galantamine or rivastigmine. A further study is needed for direct comparison of these three drugs.

Being able to predict treatment response prior to initiation of the treatment is certainly useful, but an ethical comment should be made about which treatment should be given to those patients who “a priori” are not meant to respond to treatment. The results in this study may predict responders to donepezil, but the responses to other ChEIs such as galantamine or memantine were beyond our consideration. Earlier detected nonresponders to donepezil may be promoted to be treated with other drugs.

### 5.4. Importance of Earlier Detection and Treatment

In this research field, a typical agricultural area of northern Japan, quite a few people still consider that “dementia is not a disease, but due to aging,” or “preventable by interventions such as exercise.” These misunderstandings easily prevented them from consulting doctors earlier and thus come to the memory clinic after their symptoms significantly progressed. I hope that the original questionnaire on auditory attention in daily life (Auditory ADL) helps people to detect early symptoms of AD and promote earlier treatment. Better auditory attention is considered to reduce social isolation and depression, and this could have on caregivers' burden, as they would feel listened to.

## Figures and Tables

**Table 1:** Demographics of the study population.

	*n*	Age	Men/women	Education
Young HC	15	35.7	8/7	14.5
Elderly HC	20	75.5	7/13	9.0
AD	40	79.5	16/24	8.7
(AD follow-up)	15	77.8	5/10	8.8

Means are shown for age and educational level (years).

HC: healthy control, MMSE: minimental state examination, and AD: Alzheimer's disease.

**Table 2:** Changes after donepezil treatment.

	MMSE (30)		Hearing level (dB)		ASA task				Vigilance test (33)		Auditory ADL (8–36)	
					60 dB (15)		70 dB (15)					
	Pre	Post	Pre	Post	Pre	Post	Pre	Post	Pre	Post	Pre	Post
Responders	16.5	22.5^*^	18.8	19.4	6.5	11.5^*^	5.5	6.5^*^	32.5	33.5^*^	10.5	8.5^*^
Nonresponders	15.8	16.4	17.5	18.5	6.4	6.8	5.6	5.7	31.5	30.8	9.8	10.4

MMSE: minimental state examination, ASA: auditory selective attention, ADL: activities of daily living. Parentheses indicate the maximum scores and shown are the means.

Asterisks indicated significant increase after donepezil treatment (Mann-Whitney test; *P* < 0.05).

## References

[1] Meguro M., Kasai M., Akanuma K., Ishii H., Yamaguchi S., Meguro K. (2008). Comprehensive approach of donepezil and psychosocial interventions on cognitive function and quality of life for Alzheimer's disease: the Osaki-Tajiri Project. *Age and Ageing*.

[2] Yamaguchi S., Akanuma K., Hatayama Y., Otera M., Meguro K. (2012). Singing therapy can be effective for a patient with severe nonfluent aphasia. *International Journal of Rehabilitation Research*.

[3] Shimizu S., Hanyu H., Iwamoto T., Koizumi K., Abe K. (2006). SPECT follow-up study of cerebral blood flow changes during Donepezil therapy in patients with Alzheimer's disease. *Journal of Neuroimaging*.

[4] Yoshida T., Ha-Kawa S., Yoshimura M., Nobuhara K., Kinoshita T., Sawada S. (2007). Effectiveness of treatment with donepezil hydrochloride and changes in regional cerebral blood flow in patients with Alzheimer's disease. *Annals of Nuclear Medicine*.

[5] Tateno M., Kobayashi S., Utsumi K., Morii H., Fujii K. (2008). Quantitative analysis of the effects of donepezil on regional cerebral blood flow in Alzheimer's disease by using an automated program, 3DSRT. *Neuroradiology*.

[6] Burns A., Yeates A., Akintade L. (2008). Defining treatment response to donepezil in Alzheimer's disease: responder analysis of patient-level data from randomized, placebo-controlled studies. *Drugs and Aging*.

[7] Kasuya M., Meguro K., Okamura N. (2012). Greater responsiveness to donepezil in alzheimer patients with higher levels of acetylcholinesterase based on attention task scores and a donepezil PET Study. *Alzheimer Disease and Associated Disorders*.

[8] Kasai M., Meguro K., Hashimoto R., Ishizaki J., Yamadori A., Mori E. (2006). Non-verbal learning is impaired in very mild Alzheimer's disease (CDR 0.5): normative data from the learning version of the Rey-Osterrieth Complex Figure Test. *Psychiatry and Clinical Neurosciences*.

[9] Kasai M., Ishizaki J., Ishii H., Yamaguchi S., Yamadori A., Meguro K. (2009). Normative data on Benton Visual Form Discrimination Test for older adults and impaired scores in Clinical Dementia Rating 0.5 participants: community-based study. The Osaki-Tajiri project. *Psychiatry and Clinical Neurosciences*.

[10] Meguro K., Ishii H., Yamaguchi S. (2002). Prevalence of dementia and dementing diseases in Japan: the Tajiri Project. *Archives of Neurology*.

[11] McKhann G., Drachman D., Folstein M. (1984). Clinical diagnosis of Alzheimer's disease: report of the NINCDS-ADRDA Work Group under the auspices of Department of Health and Human Services Task Force on Alzheimer's Disease. *Neurology*.

[12] Folstein M. F., Folstein S. E., McHugh P. R. (1975). ‘Mini-mental state’. A practical method for grading the cognitive state of patients for the clinician. *Journal of Psychiatric Research*.

[13] ISO. ISO 8253-1: 1989 Acoustics-audiometric test methods-part 1: basic pure tone air and bone conduction threshold audiometry.

[14] ISO (1992). Acoustics-audiometric test methods—part 2: sound field audiometry with pure tone and narrowband test signals.

[15] Nasreddine Z. S., Phillips N. A., Bédirian V. (2005). The Montreal Cognitive Assessment, MoCA: a brief screening tool for mild cognitive impairment. *Journal of the American Geriatrics Society*.

[16] Ito T., Meguro K., Akanuma K., Ishii H., Mori E. (2007). A randomized controlled trial of the group reminiscence approach in patients with vascular dementia. *Dementia and Geriatric Cognitive Disorders*.

[17] Idrizbegovic E., Hederstierna C., Dahlquist M., Nordström C. K., Jelic V., Rosenhall U. (2011). Central auditory function in early Alzheimer's disease and in mild cognitive impairment. *Age and Ageing*.

[18] Lin F. R., Metter E. J., O'Brien R. J., Resnick S. M., Zonderman A. B., Ferrucci L. (2011). Hearing loss and incident dementia. *Archives of Neurology*.

[19] Gates G. A., Gibbons L. E., McCusrry S. M., Crane P. K., Feeney M. P., Larson E. B. (2010). Executive dysfunction and presbycusis in older persons with and without memory loss and dementia. *Cognitive and Behavioral Neurology*.

[20] Nakai T., Kato C., Matsuo K. (2005). An fMRI study to investigate auditory attention: a model of the cocktail party phenomenon. *Magnetic Resonance in Medical Sciences*.

[21] Cerbai F., Giovannini M. G., Melani C., Enz A., Pepeu G. (2007). N1phenethyl-norcymserine, a selective butyrylcholinesterase inhibitor, increases acetylcholine release in rat cerebral cortex: a comparison with donepezil and rivastigmine. *European Journal of Pharmacology*.

